# Acoustic and Thermal Characterization of Therapeutic Ultrasonic Langevin Transducers under Continuous- and Pulsed Wave Excitations

**DOI:** 10.3390/s22229006

**Published:** 2022-11-21

**Authors:** Jinhyuk Kim, Jungwoo Lee

**Affiliations:** Department of Electronic Engineering, Kwangwoon University, Seoul 01897, Republic of Korea

**Keywords:** Langevin transducer, therapeutic ultrasound, pulsed excitation, heat generation

## Abstract

We previously conducted an empirical study on Langevin type transducers in medical use by examining the heat effect on porcine tissue. For maximum acoustic output, the transducer was activated by a continuous sinusoidal wave. In this work, pulsed waves with various duty factors were applied to our transducer model in order to examine their effect on functionality. Acoustic power, electro-acoustic conversion efficiency, acoustic pressure, thermal effect on porcine tissue and bovine muscle, and heat generation in the transducer were investigated under various input conditions. For example, the results of applying a continuous wave of 200 V_PP_ and a pulse wave of 70% duty factor with the same amplitude to the transducer were compared. It was found that continuous waves generated 9.79 W of acoustic power, 6.40% energy efficiency, and 24.84 kPa acoustic pressure. In pulsed excitation, the corresponding values were 9.04 W, 8.44%, and 24.7 kPa, respectively. The maximum temperature increases in bovine muscle are reported to be 83.0 °C and 89.5 °C for each waveform, whereas these values were 102.5 °C and 84.5 °C in fatty porcine tissue. Moreover, the heat generation around the transducer was monitored under continuous and pulsed modes and was found to be 51.3 °C and 50.4 °C. This shows that pulsed excitation gives rise to less thermal influence on the transducer. As a result, it is demonstrated that a transducer triggered by pulsed waves improves the energy efficiency and provides sufficient thermal impact on biological tissues by selecting proper electrical excitation types.

## 1. Introduction

The Langevin transducer, also known as the Tonpilz transducer, has widely been utilized in a variety of applications requiring high-power ultrasonic radiation. The emitted power from such transducers is used to mix and compound target materials in the sonochemistry field [1]. In medical settings, it has been shown that blood vessels may be eliminated by the transducer’s heat [2], while tumors and bones can be treated in similar ways [3,4]. Furthermore, ultrasonic devices have been applied in industrial sectors to generate heat effects on metal pieces in order to weld or remove them [5,6]. These transducers are commonly made up of several metal masses, a pair of piezoelectric rings, a pre-stress bolt, and an additional device. By tightening the central bolt, the piezoelectric components are positioned between metal masses and pre-stressed to generate acoustic power through the transducer. Additional tools like metal rods are then attached to the transducer to carry out certain surgical tasks. While electrical continuous waves are typically supplied to the piezoelectric component to maximize power output, high-power transducers are frequently driven with pulsed signals to achieve desirable results in sonochemistry [7,8], sonoluminescence [9], and industrial applications [10].

In our earlier work, we developed a transducer for therapeutic ultrasound using a MATLAB simulation of a circuit model. The Mason model and T-shaped equivalent circuit were used to reflect the complex electro-mechanical behavior of the piezoelectric material and to characterize other passive metal parts by altering their inherent attributes, such as acoustic impedance and structural features [11]. Furthermore, the bolt-clamping force was taken into account in our design procedure to predict changes in the resonance qualities of the transducer [12]. Electric matching circuits were also developed to improve transducer performance, such as acoustic power and pressure [13]. By assessing the heat effect on porcine tissue, our recent experimental results revealed that our transducer had the potential to be utilized as a medical device [14]. However, only continuous wave experiments were carried out, with no regard for energy efficiency related to heat generation in the transducer. Thus, in this work, a Langevin type transducer for tiny spot surgery was developed, and its characteristics and pressure, were evaluated by using continuous and pulse waves with varied duty factors and electrical amplitudes. Through tests, the electro-acoustic conversion efficiency was computed and connected to heat generation in the transducer, including acoustic power. Furthermore, the thermal effect induced by the pressure emitted from the transducer was investigated by measuring temperature rises in muscular and porcine tissue.

## 2. Materials and Methods

### 2.1. Fabrication of Therapeutic Langevin Transducer

**Figure 1:**
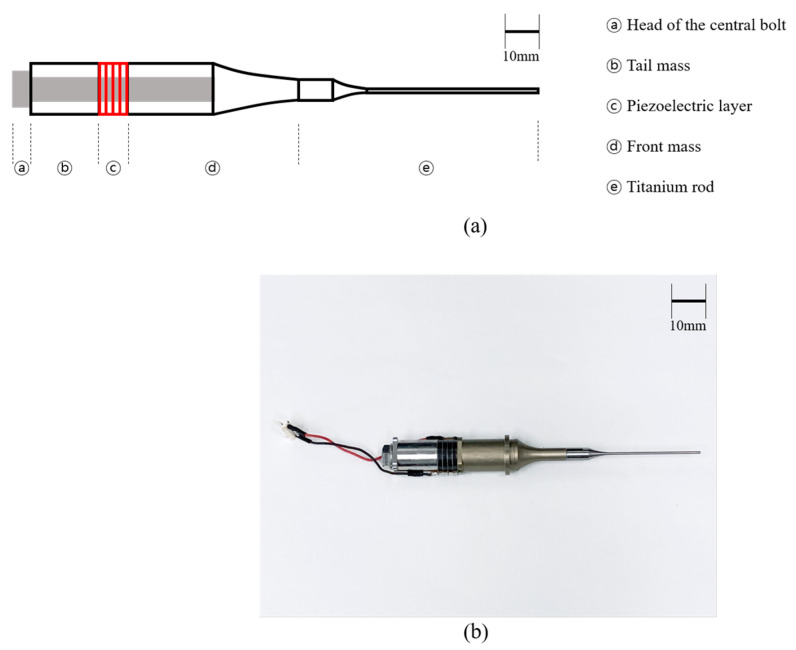
Transducer layout (**a**) cross-sectional view (**b**) actual image of the transducer.

As illustrated in Figure 1, the transducer used in this work consisted of two pairs of piezoelectric components, a central bolt, a tail mass, a front mass, and a thin rod. A central bolt with mechanical bias clamped the piezoelectric material, tail, and front masses in series. Because the Langevin type transducer has a high mechanical Q-factor, two pairs of hard type piezoelectric ceramic (C-203, Fuji Ceramic Corporation, Fujinomiya, Japan) are typically utilized. Copper-nickel electrodes of 0.2 mm in width were put in piezoelectric layers to excite the transducer. Acoustic parameters such as sound speed and density were employed to select the materials for further pieces. Materials with an acoustic impedance of 47 MRayls, such as stainless steel, are appropriate, because the tail mass must reflect the longitudinal waves from the piezoelectric layer. Aluminum is also a good front mass material, since it has a lower acoustic impedance, i.e., 17.84 MRayls, than piezoelectric elements, which show a value of 21 MRayls. An auxiliary metal rod can be added to the front mass, depending on the application. In this study, a 1.2 mm thick titanium rod was utilized in our surgical simulations. Half-wavelength synthesis theory was implemented to determine the axial length of each produced part based on the target resonance frequency, *f_r_* [11]. The target *f_r_* of the entire transducer in this investigation was set at 30 kHz, because bolt-clamped transducers are generally built to work at lower frequency ranges. In addition, because a center bolt passed through the transducer, the length had to be estimated using averaged material physical parameters, such as cross-sectional area, density, and sound velocity [11]. Table 1 summarizes the physical parameters, including dimensional features.

### 2.2. Continuous vs. Pulsed Waves

**Figure 2:**
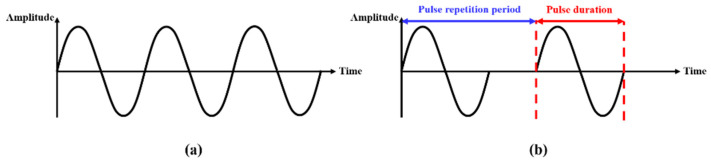
Description of waves (**a**) Continuous wave (**b**) Pulsed wave.

Depending on its state, purpose, or usability, an ultrasound transducer can be activated by an electrical continuous wave (CW) or a pulsed wave (PW). For continuous doppler mode blood flow monitoring in diagnostic applications, transducers are driven by a CW [15,16]. The transducer in power ultrasonic applications is typically powered by a continuous sinusoidal wave source, adjusted to the device’s resonance frequency [17]. A PW with a duty factor is intended to stimulate HIFU devices, i.e., imaging transducers and non-destructive detecting transducers for reflected wave reception, while also minimizing piezoelectric material fatigue [18,19,20]. In surgical settings, depending on the intended outcome, both wave conditions can activate an HIFU transducer. One study found that using a CW signal to activate an HIFU transducer resulted in a larger devascularized region than a PW signal [21]. Furthermore, continuous or pulsed ultrasound has been used not only for necrosis, but also for angionensis and vasculogenesis [22,23,24]. A graphical description of CW and PW are represented in Figure 2. The duty factor, as crucial factor of PW, is determined by Equation (1) [25]. The duty cycle in this study was controlled by the changing pulse repetition period from 3300 μs to 366 μs. The pulse duration was set to 330 μs.

(1) $$\mathrm{Duty}\;\mathrm{factor}=\frac{\mathrm{Pulse}\;\mathrm{duration}}{\mathrm{Pulse}\;\mathrm{repetition}\;\mathrm{period}}\times 100\%$$

### 2.3. Electro-Acoustic Conversion Efficiency

The electrical signal is converted to an acoustic signal, or vice versa, via an ultrasound transducer. The electro-acoustic conversion efficiency, $\eta_{eac}$, obtained using Equation (2), has been utilized to numerically describe the conversion rate of the transducer [26].

(2) $$\eta_{eac}=\frac{P_{ac}}{P_{e}}\times 100\%$$

where $P_{e}$ and $P_{ac}$ are input electrical power and output acoustic power, respectively. Notably, $P_{e}$ is determined by Equation (3), where $V_{RMS}$, I, Φ, and R are the RMS (Root Mean Square) value of peak electrical voltage, current, phase difference, and resistance, respectively. Φ is presumed to be zero because the transducer in this study is activated at a series resonance point, and I can be converted to voltage over resistance through Ohm’s equation. At resonance frequency, an impedance analyzer (4294A, Agilent, Santa Clara, CA, USA) is utilized to investigate resistance R. The calculated input energy is proportional to duty factor mentioned above.

(3) $$P_{e}=V_{RMS}\times I\times \cos\Phi =\frac{V_{RMS}{}^{2}}{R}$$

The $P_{ac}$ emitted from the transducer was measured by an ultrasound power meter (UPM-DT-1PA, Ohmic Instrument Inc., St. Charles, MO, USA). The calculated efficiency is utilized to the evaluate energy loss of the transducer under various operational conditions.

### 2.4. Characterization of Acoustic Output

**Figure 3:**
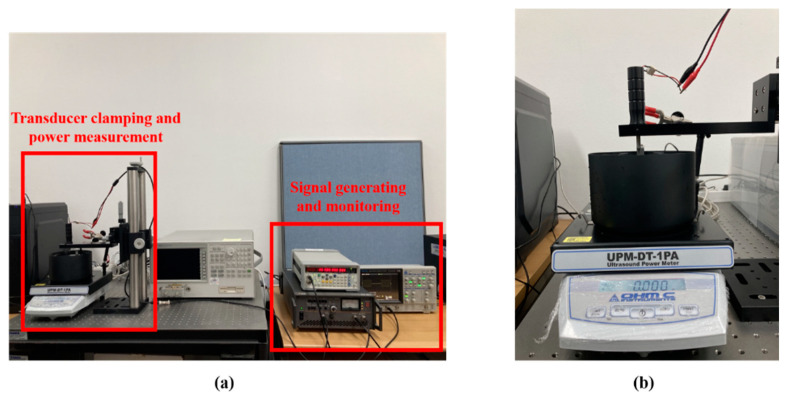
Acoustic power measurement: (**a**) whole experimental setup; and (**b**) zero-point setup of power meter.

An ultrasonic power meter was employed to evaluate the acoustic power (UPM-DT-1PA, Ohmic Instrument, St. Charles, MO, USA) of the transducer. An electrical signal was generated and amplified by a signal generator (SG382, Stanford Research System, Sunnyvale, CA, USA) and RF amplifier (HAS 4051, NF, Yokohama, Japan). The signal was monitored in real time using an oscilloscope (DS-5652, IWATSU, Kugayama, Japan). A signal generator supplied a sinusoidal signal with amplitudes ranging from 1 V peak-to-peak (V_PP_) to 2 V_PP_ at 30 kHz, augmented by an RF amplifier with a constant gain. Additionally, the transducer was excited by PW with duty factors from 10% to 90% with a 10% step and CW condition. The duty cycle was controlled by changing the pulse repetition period. The stimulated transducer radiated acoustic pressure to the target in a water bath. The emitted power was also recorded via LabView control panel. The overall setup is represented in Figure 3.

**Figure 4:**
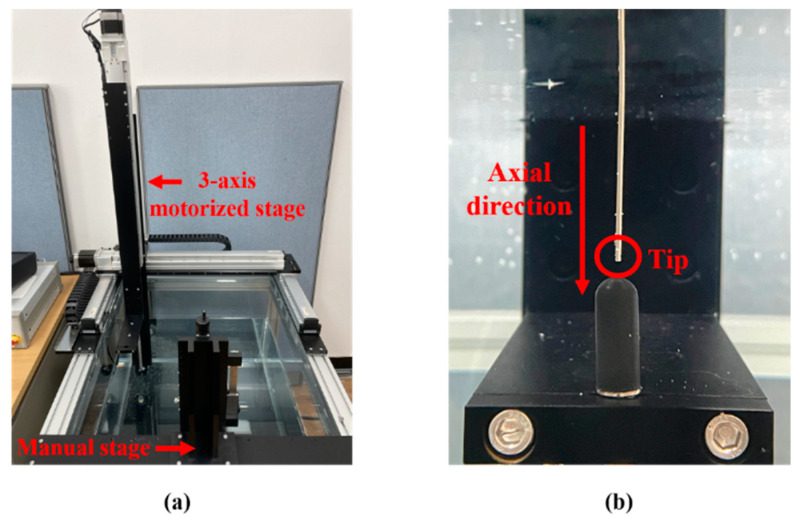
Hydrophone system: (**a**) water bath equipped with a motorized stage; and (**b**) transducer rod and hydrophone.

A hydrophone system was designed to calibrate the acoustic pressure emitted from the transducer. The system was composed of the signal generating and monitoring parts mentioned above, a hydrophone (8103, Bruel & Kjaer, Naerum, Denmark), a preamplifier (2692-0S1, Bruel & Kjaer, Naerum, Denmark), the water bath, the three-axis motorized stage and a manual stage. The hydrophone sensor was positioned at 2 mm from the tip using a motorized stage to evaluate the acoustic pressure. For the clamping transducer, the manual stage was controlled with a digital length meter. The received RF signal was pre-amplified and sent to a PC for calibration. Images of the water bath, the stages and an enlarged view of the sensor with the transducer rod are given in Figure 4. Since the diameter of the tip was 1.2 mm, i.e., much smaller than the hydrophone surface, most incident wavefronts were deflected away, with only a few being reflected back to the transducer. As a result, we assumed that the influence of standing waves on the resultant values was negligible.

### 2.5. Examination of Thermal Effect on a Biological Specimen

**Figure 5:**
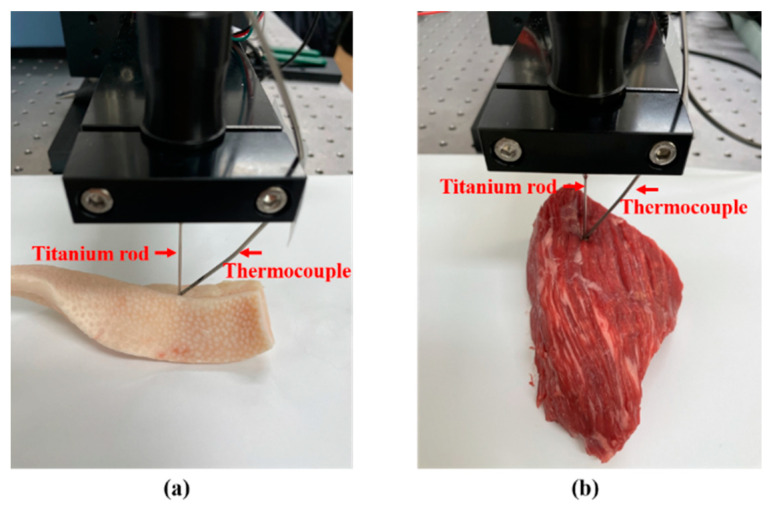
Measurement of temperature rise in tissue: (**a**) porcine fat; and (**b**) bovine muscle.

The tip of the transducer was inserted into two biological samples: porcine fat and bovine muscular tissue. Furthermore, a T-type thermocouple was implanted in those tissues to quantify the temperature rise induced by acoustic energy, which was simultaneously recorded by a data logger (EL-USB-TC, LASCAR electronics, Erie, PA, USA). The experimental setup is presented in Figure 5. We minimized the possibility of tissue properties changing because we strictly controlled their temperature with a refrigerator and a thermo-infrared camera. In addition, to avoid compounding effects due to previous trials, the target point was moved by 15 mm for each experiment, i.e., we avoided repeating the experiment in same thermal necrotized area. The depth of insertion of rod into tissue was set at 10 mm using the manual stage. We always brought the transducer to room temperature and dissipated its heat after each experiment to ensure stable performance. Furthermore, given that longitudinal waves directly transferred to the organic tissue, the thermocouple was positioned at the tip-tissue side contact surface to avoid interfering with wave propagation. The flexible thermocouple was also controlled by the manual stage.

## 3. Results

**Figure 6:**
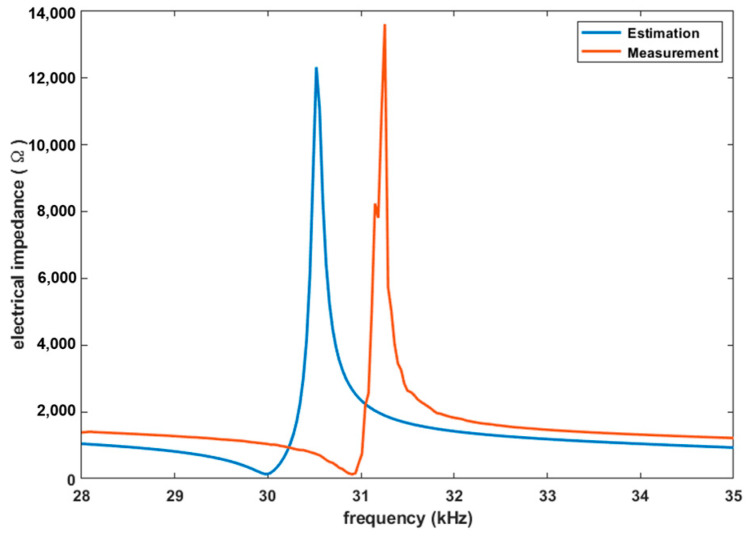
Comparison of frequency response between the experiment and the simulation.

The electrical characteristics of the transducer were investigated using an impedance analyzer (4294A, Agilent, Santa Clara, CA, USA) and the results were compared with the MATLAB simulation results. Our transducer model was analyzed using MATLAB based on an equivalent circuit [11,12,13]. In both cases, the resonance characteristics were tested between 28 and 35 kHz, as depicted in Figure 6. The simulated resonance frequency, *f_r_*, was 30 kHz with a minimum impedance of 137.19 Ω, while the anti-resonance frequency, *f_a_*, was 30.52 kHz with a maximum impedance of 12.30 kΩ. In the experimental results, *f_r_* and *f_a_* were 30.90 kHz and 31.26 kHz and the corresponding impedances were 130.74 Ω and 13.59 kΩ. There was a 0.9 kHz and a 6.55 Ω error in the resonance mode, which may have been due to a small discrepancy in the properties of the materials utilized in the simulation and the physical setup. Based on these results, pulse duration was further calculated as the integer multiple of the resonance period, yielding 30.9 kHz.

**Figure 7:**
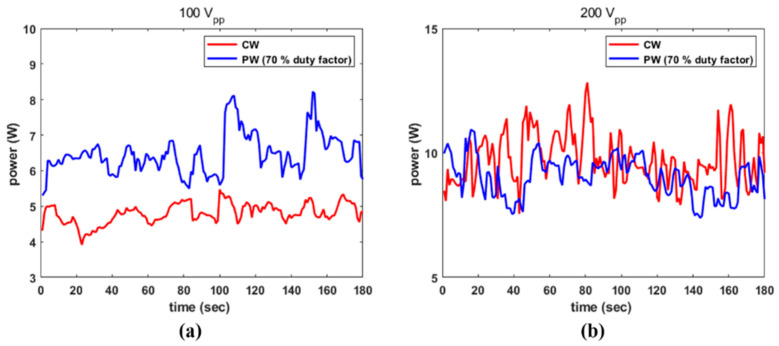
Acoustic power depending on CW and PW conditions: (**a**) 100 V_PP_; and (**b**) 200 V_PP_.

The acoustic power radiating from the transducer excited by CW and PW was examined. The transducer was excited by PW in 10% increments from 10% to 40%, but no meaningful output could be measured. With a duty factor of 50% or above, the transducer was capable of transmitting enough acoustic pressure to be probed. For instance, the radiated power from the transducer stimulated by CW with 100 V_PP_ and 200 V_PP_ and by a PW with a 70% duty factor with same amplitude is compared in Figure 7. For the former, CW produced 4.80 W of temporal averaged power (P_temp_) over 180 s and while PW radiated 6.46 W under same conditions. $\eta_{eac}$, calculated using Equations (2) and (3) was 12.55% and 24.13%, respectively. Furthermore, at 200 V_PP_, CW and PW yielded a P_temp_ of 9.73 W and 9.03 W, respectively, corresponding 6.40% and 8.43% efficiency. As Equation (3) shows, a high electrical field supplied to the transducer increased the dielectric loss, leading to a reduction in $\eta_{eac}$.

**Figure 8:**
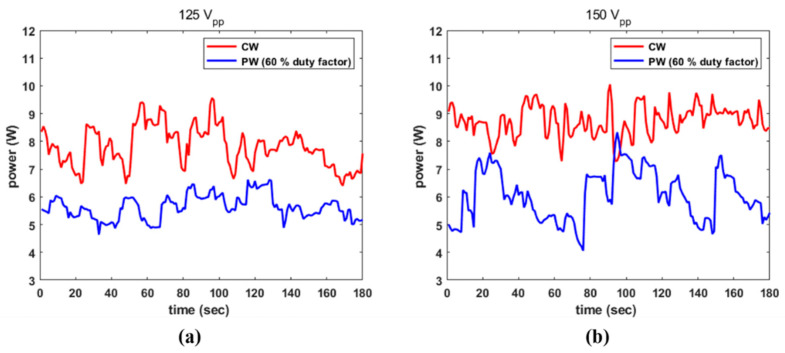
Emitted power from the transducer by CW and PW with 60% duty factor: (**a**) 125 V_PP_; and (**b**) 150 V_PP_.

CW with 125 V_PP_ and 150 V_PP_ and a 60% duty factor with PW at the same amplitude were examined to study different electrical scenarios. The transducer stimulated by CW and PW with 125 V_PP_ emitted a P_temp_ of 7.81 W and 5.63 W, respectively, as illustrated in Figure 8a. In these instances, $\eta_{eac}$ was 13.07% and 15.71%, respectively. The same datasets, as shown in Figure 8b were 8.74 W, 6.07 W, 10.16%, and 11.76%. The efficiency of the transducer under a strong electric field was also diminished in these cases.

The temporal averaged power and $\eta_{eac}$ of the transducer under all conditions are summarized in Table 2. In general, efficiency decreased with higher electrical amplitude, owing to dielectric loss proportional to the applied electric field. The lowest efficiency, i.e., 6.40%, was found with CW at 200 V_PP_.

**Figure 9:**
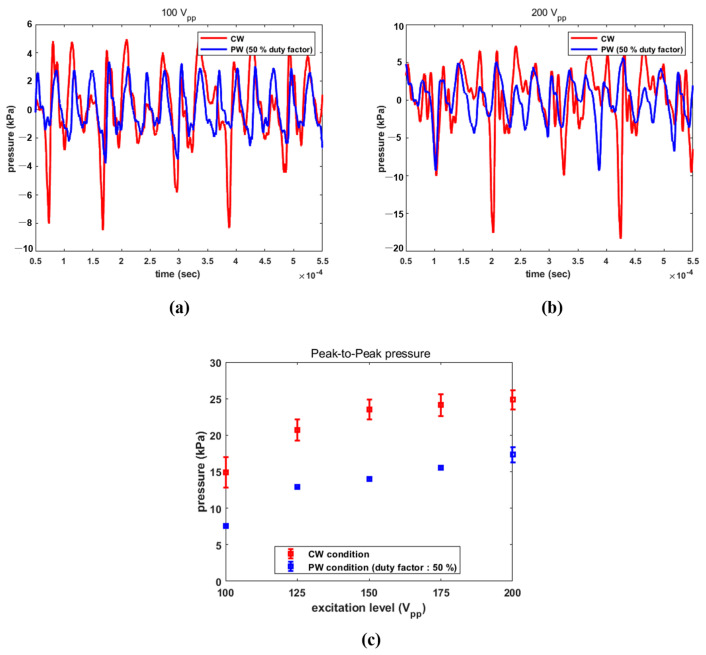
Calibration data of acoustic pressure under CW and PW with a 50% duty factor: (**a**) 100 V_PP_; (**b**) 200 V_PP_; and (**c**) comparison of peak-to-peak pressure with standard deviation.

The calibrated acoustic pressure data are plotted in Figure 9. Though the pressure from surface of the tip needs to be further explored, a 2 mm spacing was established to minimize direct damage to the hydrophone sensor due to acoustic cavitation. All electrical conditions were the same as in the previous experiment. The transducer activated by CW with 100 V_PP_ emitted positive and negative peak pressures of 5.10 kPa and −8.49 kPa, respectively, with a peak-to-peak pressure of 13.59 kPa. For PW with 100 V_PP_, the corresponding values were 3.39 kPa, −3.8 kPa and 7.19 kPa. In addition, as the applied electrical amplitude was increased, positive and negative pressure also increased, as seen in Figure 9a,b. The transducer radiated positive and negative pressures of 7.45 kPa and −18.33 kPa, respectively, in the CW scenario. The PW signal led the transducer to produce 5.58 kPa and −9.27 kPa under the same conditions. Their respective peak-to-peak pressures were 25.78 kPa and 14.85 kPa. Figure 9c indicates the peak-to-peak pressures with standard deviation from the transducer under the above-mentioned conditions. These figures were determined by averaging the calibrated pressures from five separate trials. The acoustic pressure was proportionate to the applied electrical amplitude in both situations. A maximum value of 24.84 kPa was observed at CW with 200 V_PP_, while the smallest value of 7.55 kPa was obtained at PW with 100 V_PP_.

**Figure 10:**
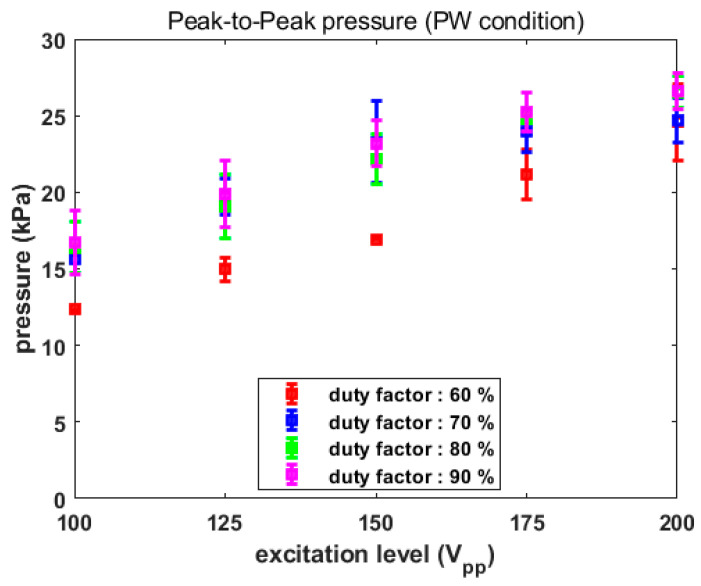
Acoustic pressure under various PW conditions.

Similarly, the computed peak-to-peak pressure under different conditions is reported in Figure 10, where the averaged values were proportional to electrical amplitude, as in the previous case. Though there were two exceptions at 70% duty factor; at the same amplitude, as the duty factor was raised, the emitted pressures generally increased. Experiments were repeated five times under various PW conditions in order to identify acoustic radiation pressures that were similar to the CW results. For the case of a 70% duty factor, the computed averaged pressure was 15.62 kPa, 19.68 kPa, 23.27 kPa, 23.94 kPa and 24.7 kPa, sequentially. Since these values had minimal differences from those CW results in terms of pressure, experiments using PW with a 70% duty factor and CW were undertaken to compare the thermal effect on biological tissue.

**Figure 11:**
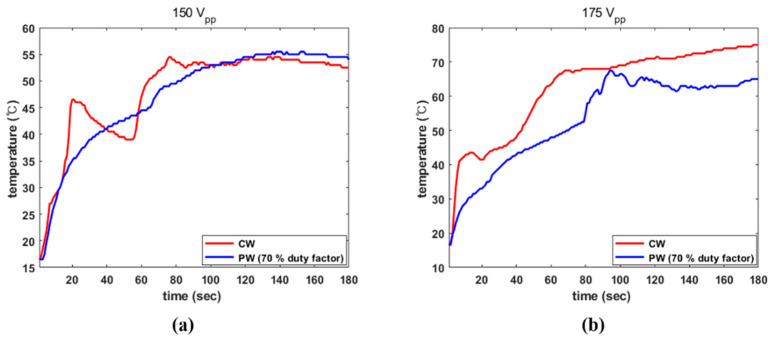
Comparison of temperature increases induced by different electrical conditions: (**a**) 150 V_PP_; and (**b**) 175 V_PP_.

In addition, thermal ablation caused by acoustic pressure was identified by investigating increases in temperature in our tissue specimens. Figure 11 demonstrates an increasing trend in temperature in the muscle tissue under various operational conditions. With CW excitation, dramatic rising trends were detected in both instances at an early stage. As shown in Figure 11a, while temperature reached 46.5 °C in 20 s under CW conditions, PW excitation made the temperature increase to 35 °C. The maximum temperatures induced by CW and PW at 150 V_PP_ were 54.5 °C and 55.5 °C, respectively. As shown in Figure 11b, the CW signal led to a temperature rise of 42.5 °C in only 10 s, whereas PW achieved the same temperature in 38 s. The maximum recorded temperatures were 75 °C and 67.5 °C.

**Figure 12:**
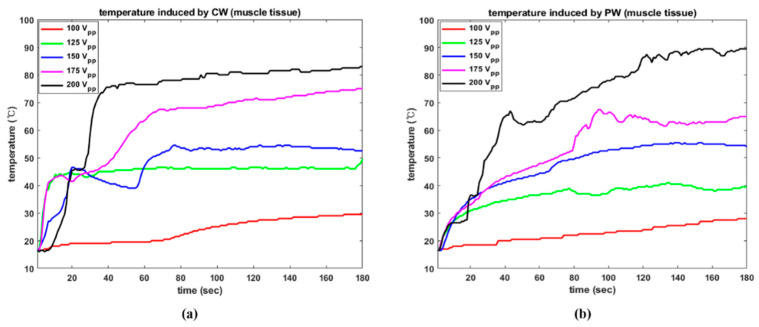
Temperature changes in bovine muscle as a function of various electrical driving conditions. (**a**) CW (**b**) PW.

The temperature responses under all setup conditions are given in Figure 12. The results are based on trial number 1. The electrical amplitude of 100 V_PP_ was insufficient to observe a meaningful thermal effect on the tissue in both situations. Except in these instances, CW excitation resulted in a significant temperature rise in the early stages when compared to PW results using the same electrical amplitude. For 200 V_PP_, CW and PW induced maximum temperatures of 83.0 °C and 89.5 °C, respectively. In the CW condition, it took 34 s to achieve a temperature of 71 °C, but in the PW condition, this required 77 s. Similarly, the highest temperatures caused by 125 V_PP_ were 49.5 °C and 41 °C. In this example, again, CW needed only 10 s to reach 41.5 °C but PW required 122 s to exceed 40 °C.

**Figure 13:**
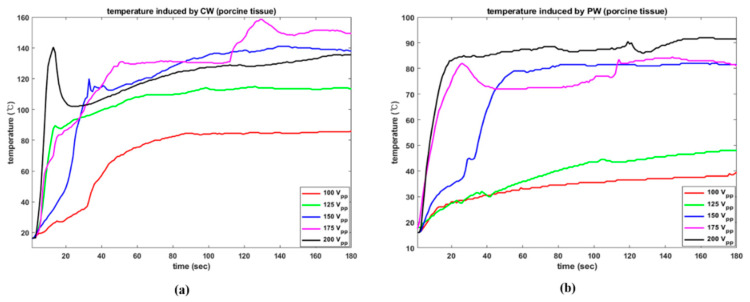
Temperature rises in porcine tissue over time; (**a**) CW; and (**b**) PW.

Furthermore, the temperature was monitored while the emitted acoustic pressure was being applied to porcine tissue. Regardless of the electrical stimulation settings, the observed maximum temperatures were consistently higher than those of the smuscle tissue. The fat component dissolved by acoustic pressure reacted violently, causing it to boil. The highest temperature increases with CW were 86 °C, 115 °C, 141 °C, 158.5 °C, and 140.5 °C, according to the electrical amplitude. The same dataset for PW was 39.5 °C, 48 °C, 82 °C, 84.5 °C, and 92 °C. During the first 30 s, the CW stimulation induced temperatures of 35 °C, 95 °C, 104 °C, 99 °C, and 102.5 °C, depending on electrical amplitude, whereas PW induced temperatures of 29 °C 30 °C, 45 °C, 79.5 °C, 84.5 °C. These rapid temperature increases with CW were identical to those seen in the muscle tissue test. According to the results of the bovine muscle and porcine tissue tests, excitation conditions leading to temperature increases of between 60 °C and 95 °C could be employed to induce tissue coagulation and necroses of tumors, [27] cancer cells [28,29] or muscle [30]. Temperatures generally did not increased monotonically because acoustic cavitation caused microbubble collapse which induced shock waves, causing high temperatures. Abundant microbubbles in the early phases of each experiment resulted in rapid, violent, and nonlinear increases in temperature. All experimental results were represented in Figure 13.

Overall, regardless of the tissue type, CW produced sudden increases in temperature, whereas PW generated steady increases which were demonstrated to be more controllable. PW excitation was deemed to be appropriate because of the moderate temperature increase, i.e., comparable to that of radiofrequency (RF) ablation, because the transducer was intended to treat sensitive and tiny locations, such as the thyroid [31]. However, considering low temperature elevations such as those observed in the muscle tissue experiments with 100 V_PP_, there is a demand for Langevin transducers to use high-power ultrasound without temperature increases [32,33].

**Figure 14:**
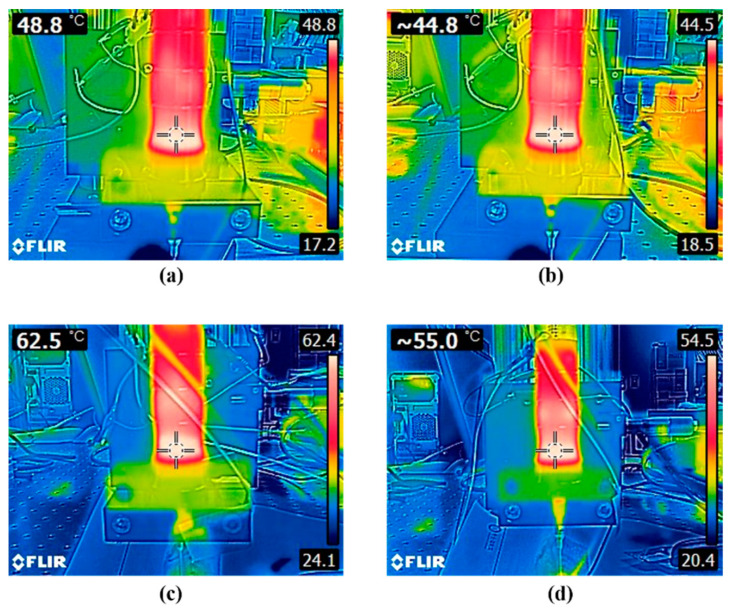
Heat generation in the piezoelectric element under CW and PW excitations. Top row: bovine muscle (**a**) with CW 175 VPP and (**b**) with PW 175 VPP. Bottom row: porcine fat (**c**) with CW 200 VPP and (**d**) PW 200 VPP.

The input energy to the transducer is not transmitted completely to the output signal during the conversion process, resulting in heat dissipation in the transducer structure, particularly in the piezoelectric stacks. To assess heat generation as a result of such losses, the temperature of the piezoelectric component was measured immediately after each trial using a thermo-infrared camera (E5-XT, FLIR, Wilsonville, OR, USA). In the muscle tissue test, a temperature of 48.8 °C was observed at the piezoelectric component after operation using CW with 175 V_PP_, whereas PW with the same amplitude generated a temperature of 44.8 °C. At 200 V_PP_ in the porcine tissue experiment, CW and PW produced temperatures of 62.5 °C and 55.0 °C, respectively. Figure 14 shows the captured thermal camera images.

In general, CW stimulation caused more heat in the transducer than PW due to increased dielectric loss, which was proportional to higher electric energy. The temperature readouts at the piezoelectric element under CW and PW excitations are summarized in Table 3.

## 4. Conclusions

This work investigated the influence of electrical excitation conditions on the operation of a Langevin transducer. Acoustic power, electro-acoustic conversion efficiency, acoustic pressure, temperature in bovine muscle and porcine fat, and heat generation at the transducer were measured to evaluate its acoustic and thermal characteristics. To this end, we developed a transducer model for therapeutic ultrasound and excited the device at the resonance frequency, as determined by an impedance analyzer. Pulsed waves with variable duty factors were designed to assess the operation of the transducer depending on the electrical conditions. The results showed that pulse-driven transducers might be suitable for surgical procedures with sensitive tissue treatments, achieving lower heat generation near the transducer due to better conversion efficiency. Therefore, this study verifies the suitability of Langevin transducers for the thermal treatment of various tissues when the electrical excitation parameters are closely controlled.

## Figures and Tables

**Table 1:** Physical and dimensional characteristics of the transducer.

Part	Material ^3^	Sound Speed (m/s)	Density (kg/m^3^)	Acoustic Impedance (MRayls)	Axial Length (mm)	Inner Diameter (mm)	Outer Diameter (mm)
Central bolt	Stainless steel	5920	8000	47	55	-	6.5
Tail mass	Stainless steel	5920	8000	47	20	6.5	15
Piezoelectric element	Hard ceramic	2791	7700	21	8	6.5	15
Front mass	Aluminum	6375	2700	17	50	6.5	15 ^1^
Auxiliary rod	Titanium	4987	4430	22	60	-	6 ^2^

^1^ The front mass, including the exponential-shaped booster, had a large end and a small end. The outer diameters of the large and small ends were 15 mm and 6 mm, respectively. ^2^ The surgical rod also included a horn-shaped structure. The diameter of the tip of the rod was 1.2 mm. ^3^ The material properties are described in [12].

**Table 2:** Averaged acoustic power (W) and electro-acoustic conversion efficiency (%) of the transducer.

	Amplitude (V_PP_) ^1^	100	125	150	175	200
Duty Factor (%) ^2^						
50		2.40 W/12.55%	4.14 W/13.86%	5.51 W/12.81%	6.24 W/10.66%	6.90 W/9.02%
60		3.26 W/14.21%	5.63 W/15.71%	6.07 W/11.76%	8.36 W/11.90%	8.97 W/9.77%
70		6.46 W/24.13%	7.71 W/18.43%	8.17 W/13.56%	8.90 W/10.86%	9.03 W/8.43%
80		6.28 W/20.53%	7.83 W/16.38%	8.58 W/12.46%	8.87 W/9.50%	9.71 W/7.93%
90		6.33 W/18.39%	7.84 W/14.58%	8.58 W/11.08%	8.90 W/8.44%	9.82 W/7.13%
CW		4.80 W/12.55%	7.81 W/13.07%	8.74 W/10.16%	8.95 W/7.64%	9.73 W/6.36%

^1^ Electrical amplitude varying from 100 V_PP_ to 200 V_PP_ with 25 V_PP_ increments. ^2^ The duty factor was changed from 50% to 90% with 10% increments.

**Table 3:** Temperature at the piezoelectric element in each tissue test.

CW	100 V_PP_	125 V_PP_	150 V_PP_	175 V_PP_	200 V_PP_
Bovine muscle	34.2 °C	34.5 °C	43.2 °C	48.8 °C	51.3 °C
Porcine tissue	34.8 °C	34.9 °C	42.7 °C	50.4 °C	62.5 °C
PW (70% duty factor)	100 V_PP_	125 V_PP_	150 V_PP_	175 V_PP_	200 V_PP_
Bovine muscle	32.8 °C	33.8 °C	39.5 °C	44.8 °C	50.4 °C
Porcine tissue	33.3 °C	33.9 °C	35.1 °C	44.9 °C	55.0 °C
